# *usp*A gene-based phylogenetic analysis and antigenic epitope prediction for *Escherichia coli* strains of avian origin

**DOI:** 10.3389/fvets.2023.1183048

**Published:** 2023-12-21

**Authors:** Kushal Grakh, Dinesh Mittal, Anand Prakash, Ramesh Kumar, Naresh Jindal

**Affiliations:** Department of Veterinary Public Health and Epidemiology, Lala Lajpat Rai University of Veterinary and Animal Sciences, Hisar, India

**Keywords:** B-cell, *E. coli*, epitope, peptide, *Shigella*, UspA

## Abstract

Pathogenic *Escherichia coli* (*E. coli*) is responsible for various local and systemic infections in animal and human populations. Conventional methods for the detection and identification of *E. coli* are time-consuming and less reliable for atypical strains. The *uspA* gene has been widely used as a target for the detection of *E. coli*. The present study was aimed at phylogenetic analysis of the *uspA* gene sequences to determine the evolutionary relationships between the strains and other members of the *Enterobacteriaceae* family. In addition, the unique differences in the sequences of the current study with *Salmonella* and *Shigella* species were tested using Tajima’s molecular clock test. Antigenic epitope prediction was performed to locate the B-cell epitope region of the UspA protein. Two *E. coli* isolates of avian origin and strains from the National Center for Biotechnology Information (NCBI) database were used for prediction. The Immune Epitope Database (IEDB) server, Bepitope, ABCpred, SVMTrip, and ElliPro server were used to identify B-cell epitopes. The 3D structure was predicted using SWISS-MODEL. Phylogenetic analysis of the isolates from the current study revealed that both OM837340 and OM837341 sequences from the current study had maximum nucleotide homology (nt) of 99.87%–100% with *E. coli* isolates and minimum nt homology of 84.08% with *Salmonella enteritidis* and *S*. Hissar. The isolates in the current study had a homology of 98.87%, while the homology with *Shigella* species was 99.25%. Seven silent mutations were observed in the coding region of the UspA protein of ECO9LTBW (current study). Modeling of the UspA protein revealed a maximum homology of 67.86% with the Protein Data Bank in Europe (PDBe), also validated by the Ramachandran plot. No significant differences were found in the coding regions of *usp*A of *Salmonella*, *Shigella*, and *E. coli* with Tajima’s test. For the *E. coli* isolates, a total of 24 linear B-cell and seven discontinuous epitopes were predicted using *in-silico* analysis. When the results of the predicted peptides were compared, two peptides, namely ARPYNA and YSDLYTGLIDVNLGDMQKRISEE, were found suitable candidates. In conclusion, the *usp*A gene appears to be conserved among *E. coli* isolates and can be used for molecular detection.

## 1 Introduction

*Escherichia coli* (*E. coli*) is a diverse group of bacteria belonging to the *Enterobacteriaceae* family and is associated with various diseases in humans and animals. *E. coli* is responsible for septicemia, peritonitis, meningitis, abscesses, and urinary tract infections (UTI) in humans (1). In animals, *E. coli* is associated with a variety of infections including metritis, mastitis, septicemia, neonatal diarrhea, and UTI (2). Avian pathogenic *E. coli* (APEC) causes avian colibacillosis responsible for huge economic loss to the poultry industry (2). The 77 min-long *E. coli* chromosome contains the 435 bp-long *usp*A gene, which encodes the UspA protein (positions 54,079 to 54,878, GenBank accession number U00039).

The genomes of bacteria, fungi, archaea, protozoa, and plants all have a conserved set of proteins belonging to the superfamily of universal stress protein (Usp) (3, 4), but most of their biochemical roles and biological functions remain unknown (3). Survival of *E. coli* during cell growth, adhesion, and motility depends on the *uspA* gene, which is one of the six *usp* genes found in this organism (4). The *uspA* gene has been used worldwide to identify and confirm *E. coli* (2, 5–7). Stressors like heat shock, nutrient depletion, osmotic pressure, toxic substances, etc. promote the synthesis of Usp protein (8).

Peptide-based diagnostic tools are increasingly used for both human and animal diseases (9). If point-of-care diagnostics is available for treating certain infections, swift intervention and therapy could be possible. Further research and studies are needed for the development of peptide-based diagnostic tools, given the wide variety of pathogenicity and pathogenicity characteristics of APEC.

In the present work, *uspA* gene sequencing and phylogenetic analysis were used to characterize *E. coli* isolates grown from chickens with colibacillosis and their environment. To propose a peptide-based diagnostic test or vaccination, the identification of B-cell epitopes (antigenic regions that activate B-cell response) is a crucial first step. The u*sp*A gene was chosen as it is conserved among different *E. coli* strains and is widely used to identify *E. coli* using PCR making it an ideal target for antigenic epitope studies in these bacteria (10). In addition, the gene can elicit an immune response and is critical for bacterial survival because of its involvement in stress response pathways (3).

Several online resources are accessible for the prediction of discontinuous and linear (3D or continuous in sequence) antigenic epitopes (11). The main goal of the current work is to phylogenetically analyze the *usp*A gene, model the UspA protein, and then predict the antigenic epitope of the protein to better understand UspA and provide a basis for further research. This information will be used as a basis for future applied research.

## 2 Materials and methods

### 2.1 Source of samples

The samples used in the current study were from our previous study (2). Two avian-origin *E. coli* isolates viz., APEC41LFB isolated from the liver of the colibacillosis-affected (dead) bird and ECO92LTBW isolated from litter material of poultry shed were used. Details of the isolates used in the current study, including consent and ethical approval requirements can be found in the previous study (2).

### 2.2 Sequencing and phylogenetic analysis

The PCR product, i.e., *usp*A gene (884 bp), was amplified using primers described by Osek (6). The PCR products were purified using a Gel extraction kit (Qiagen, Germany), and *usp*A-specific primers were used as the sequencing primers (AgriGenome Labs Pvt. Ltd., India). The sequences were then submitted to GenBank and accession numbers were assigned. The ClustalW algorithm was used in the MEGA 11.0 program to align the nucleotide (nt) sequences of *uspA* with other sequences acquired from the NCBI database. The evolutionary relationship was deduced by building a phylogenetic tree using the maximum likelihood approach, the Kimura-2 parameter (K-2) model, complete deletion for missing data, and a rate difference of five categories in MEGA 11.0 (12, 13). Tajima’s molecular clock theory was examined to determine if there was any notable divergence between the sequences (14). After nucleotide alignment, the deduced amino acid sequences were captured and further processed. BioEdit was used to generate the dot plot for the nucleotides within the coding region (15).

### 2.3 Molecular modeling and protein structure assessment

The homology-modeling server SWISS-MODEL (16), was used for modeling of UspA protein. A three-dimensional model for UspA protein was created utilizing the list of 48 templates. The geometrical properties of the modeled protein structures were evaluated using qualitative model energy analysis (QMEAN). The models with the greatest QMEAN values were selected. For structural validation, the Ramachandran plot for the models was generated using MolProbity (17).

### 2.4 Antigenic epitope prediction

The web-based Immune Epitope Database Analysis Resource (IEDB) tool was used to predict B-cell epitope for translated ORF region of the *usp*A gene (accessed at: http://tools.immuneepitope.org/tools/bcell/iedb input). The methods used by the tool included Chou and Fasman beta turn prediction (18), Emini surface accessibility prediction (19), Karplus and Schulz flexibility prediction (20), Kolaskar and Tongaonkar antigenicity prediction (21), Parker hydrophilicity prediction (22), BepiPred linear epitope prediction (23), and BepiPred linear epitope prediction 2.0 (24). Standalone servers ABCpred (25) and SVMTrip (26) were also used for antigenic epitope prediction.

### 2.5 Peptide comparison and selection

B-cell epitopes predicted by different tools were analyzed based on the scores of four models, namely the antigenicity method of Kolaskar and Tongaonkar, the Emini surface accessibility prediction method, the beta-turn method of Chou–Fasman, and the hydrophilicity method of Parker (27, 28). The peptides with scores above the threshold for each method were selected as the most suitable candidates.

### 2.6 Structure-based epitope prediction

The online tool ElliPro (found at: http://tools.immuneepitope.org/tools/ElliPro/iedb input) was used to predict discontinuous epitopes from 3D structures (pdb format) of proteins based on solvent accessibility and flexibility (29). The input files for the APEC41LFB were sent separately to the server in pdb format, with the lowest value set to 0.7 and the maximum distance set to 6 Å.

### 2.7 Ethical statement

For this study strains from our previous study (2) were used. Therefore, approval was not required from the Institutional Ethics Committee.

## 3 Results

Two *E. coli* isolates (one pathogenic and one non-pathogenic) were used in the current study. Isolation and identification of *E. coli* isolates used in the study were based on cultural, morphological, VITEK2, and *usp*A gene amplification by PCR. The resulting amplicon of 884 bp, and was further sequenced by Sanger sequencing. The nucleotide sequences of the *usp*A genes from pathogenic (APEC41LFB) and non-pathogenic (ECO92LTBW) *E. coli* were deposited at GenBank and assigned accession numbers OM837340 and OM837341, respectively.

### 3.1 Phylogenetic analysis

**Figure 1:**
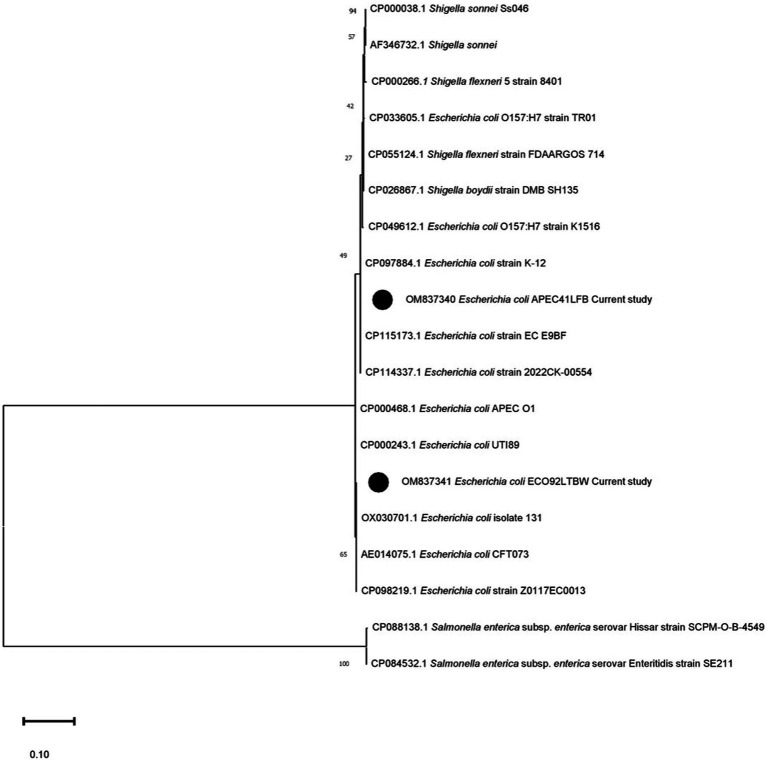
Phylogenetic analysis using the *uspA* gene’s nucleotide sequences. The phylogenetic relationship was established using MEGA 11.0 software with the maximum likelihood method and the Kimura-2 parameter (K-2) model. Texas lists the GenBank accession numbers of the compared strains. Each tree node displays the bootstrap values (%). Black circles denote the present research strains (OM837340) and (OM837341).

**Figure 2:**
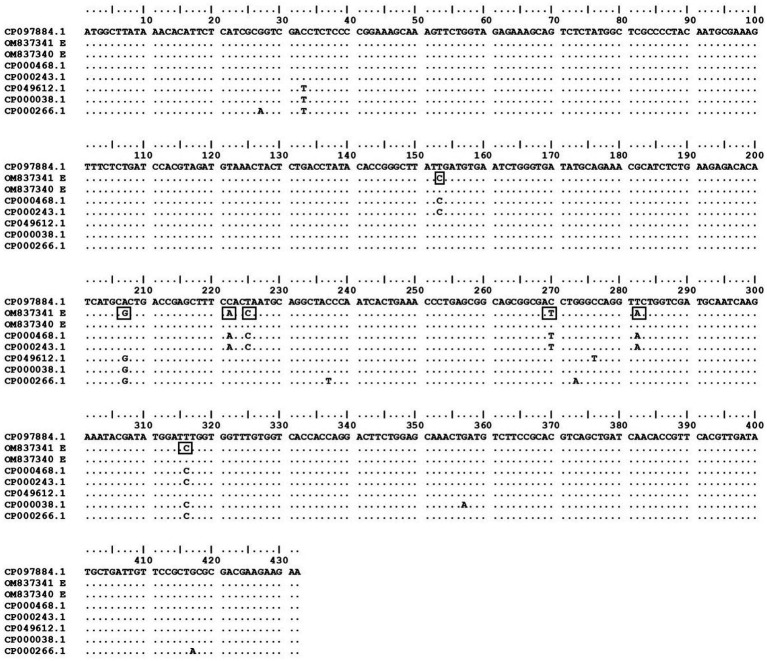
Dot plot of coding region of current study isolates with other strains from NCBI constructed using BioEdit version 7.0. *E. coli* strain K-12 MG1655 was used as the standard strain to detect any changes in the nucleotide bases. The changes in the bases of current study sequence OM837341 are presented in rectangular boxes.

The sequenced information of OM837341 and OM837340 was analyzed for phylogeny and compared with the existing databases of various *E. coli* strains and other species to determine possible changes in nucleotide and amino acid composition. The sequence OM837341 (non-pathogenic isolate) showed a maximum of 100 percent nucleotide (nt) homology with *E. coli* sequences CP098219.1, AE014075.1, and OX030701.1, whereas a minimum nt homology of 85% was observed with *Salmonella enteritidis* and *S*. Hissar sequences (CP084532.1 and CP088138.1). Sequence OM837340 (pathogenic isolate) revealed a maximum homology of 99.87% with *E. coli* (CP115173.1 and CP097884.1) and a minimum of 84.75% homology with *S. enteritidis* and *S*. Hissar (CP084532.1 and CP088138.1). The two strains in the current study showed homology of 98.87%–99.25% with *Shigella* species (Figure 1). Seven silent mutations were observed in the nucleotide sequence OM837341 when compared to the standard *E. coli* K-12 sequence (CP097884.1) (Figure 2).

### 3.2 Tajima’s molecular clock hypothesis test

The test of Tajima’s molecular clock hypothesis was performed for sequence A (OM837341) and sequence B (OM837340) with sequence C (CP084532.1 *Salmonella enterica* subsp. *enterica* serovar Enteritidis) and sequence D (CP055124.1 *Shigella flexneri*) used separately to determine evolutionary divergence in the coding region of *uspA* gene. With *S. enteritidis* serving as the outgroup, 297 identical sites, three divergent sites, two distinct differences in sequences A and B, and 390 in sequence C were detected (*p* = 1.00). When *Shigella* was used as an outgroup, 785 identical sites, 0 divergent sites, 5 unique differences in sequence A, 3 unique differences in sequence B, and 2 unique differences in sequence D were detected (*p* = 0.479). The null hypothesis of an equal rate in the different lineages was not rejected.

### 3.3 Protein structure

**Figure 3:**
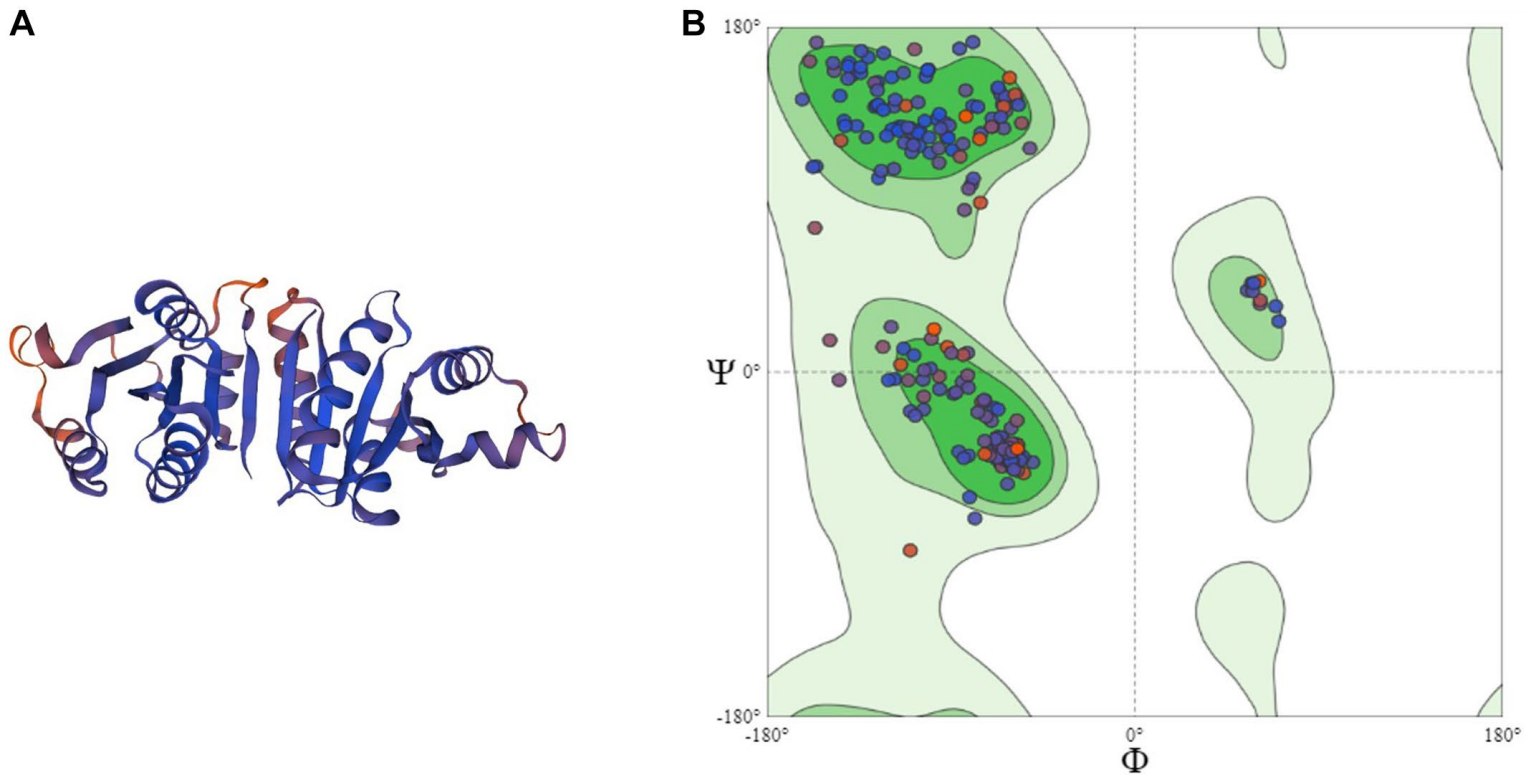
**(A)** 3D structure of UspA protein and **(B)** Ramachandran plot of UspA protein.

Homology modeling of UspA protein using the SWISS Model revealed a maximum identity of 67.86% for the current study protein with Protein Data Bank in Europe (PDBe): 1jmv chain A (x-ray diffraction 1.85 Å). The server modeled the structure with 99.92% confidence using the homology and the PDBe: 1jmv chain A as a template (Figure 3A). A Ramachandran plot was generated and no unexplained outliers were found. This indicates that the structure was of high quality (Table 1). The QMEAN score was −0.73. The Ramachandran plot showed that the modeled structure had a 97.05% favorable zone, which served as validation (Figure 3B).

**Table 1:** Results of the Ramachandran analysis using MolProbity.

MolProbity score	1.31	
Ramachandran favoured	97.05%	
Clash score	3.50	(A67 HIS-A86 SER)
Ramachandran outliers	0.0%	
Rotamer outliers	0.82%	A22 LYS, B111 HIS
C-beta deviations	1	A90 ASP
Bad bonds	0/2,176	
Bad angles	35/2,957	B43 TYR, (B28 ARG-B29 PRO), A104 MET, (A28 ARG-A29 PRO), B133 ASP, A105ASP, B105 ASP, (A79 TYR-A80 PRO), (B13 SER-B14 PRO), A112 HIS, A90 ASP, B25SER, B68 HIS, A38 HIS, B57 ASP, B104 MET, B67 HIS, A68 HIS, B122 SER, A42ASN, B111 HIS, A131 HIS, A111 HIS, A25 SER, B5 HIS, A114 ASP, B13 SER, B112HIS, A62 ILE, A5 HIS, B38 HIS
Cis prolines	1/265	(B43 TYR-B44 SER)

### 3.4 Target UspA protein sequence and epitope prediction

**Figure 4:**
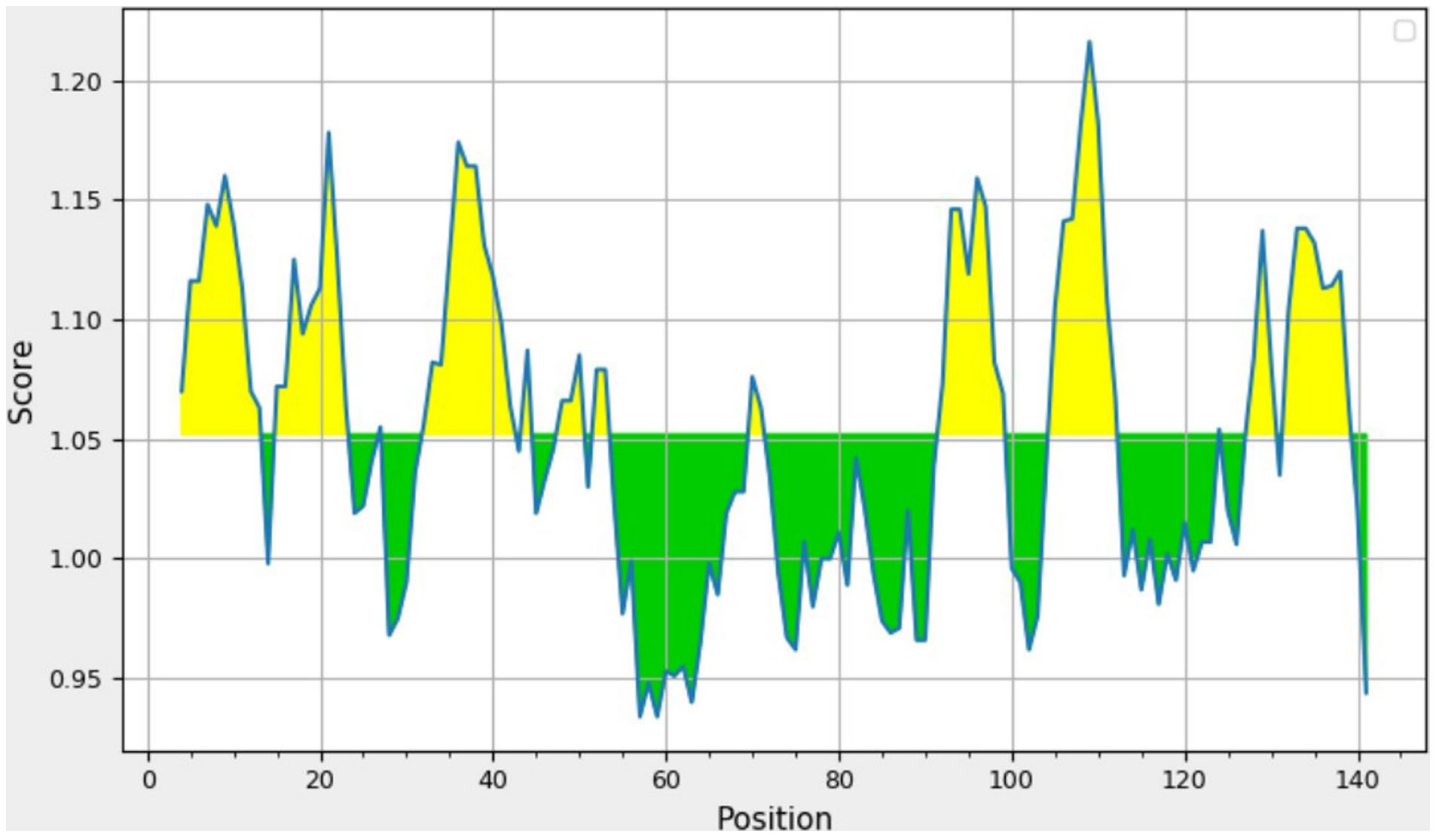
Graphical representation of the results predicted by Kolaskar and Tongaonkar antigenicity method [Threshold (Th) = 1.052]. The area in yellow depicts the score above the threshold and the green depicts the score below the threshold value.

The consensus sequence was generated by aligning different retrieved sequences using BioEdit software. The sequence obtained was 144-mer in length: “MAYKHILIAVDL SPESKVLVEKAVSMARPYNAKVSLIHVDVNYSDLYTGLIDVNLGDMQKRISEETHHALTELSTNAGYPITETLSGSGDLGQVLVDAIKKYDMDLVVCGHHQDFWSKLMSSARQLINTVHVDMLIVPLRDEEE.” Similarity between the current study strains and 100 other sequences available in NCBI was examined using the basic local alignment search tool protein (BLASTp). Since the protein sequences of the all the compared isolates were completely identical, additional parameters could be inferred from any single sequence. According to the method of Kolaskar and Tongaonkar, the 144 amino acid sequence contained six antigenic peptides. The antigenic peptides had three octapeptides ranging from 8 to 11 amino acids in length. Table 2 lists the peptide lengths, their sequences, and their position along the entire length of the sequence. Figure 4 illustrates the anticipated peptides of *E. coli* isolates based on antigenic propensity (*y*-axis) and sequence position (*x*-axis). The average antigenic propensity value was 1.052, with minimum and maximum ranging from 0.934 to 1.216, respectively.

**Table 2:** Predicted antigenic epitope peptides of *E. coli* for UspA protein with Kolaskar and Tongaonkar antigenicity method.

No.	Start position	End position	Peptide	Peptide length
1	4	13	KHILIAVDLS	10
2	15	23	ESKVLVEKA	9
3	32	42	AKVSLIHVDVN	11
4	92	99	GQVLVDAI	8
5	105	112	DLVVCGHH	8
6	132	139	VDMLIVPL	8

**Figure 5:**
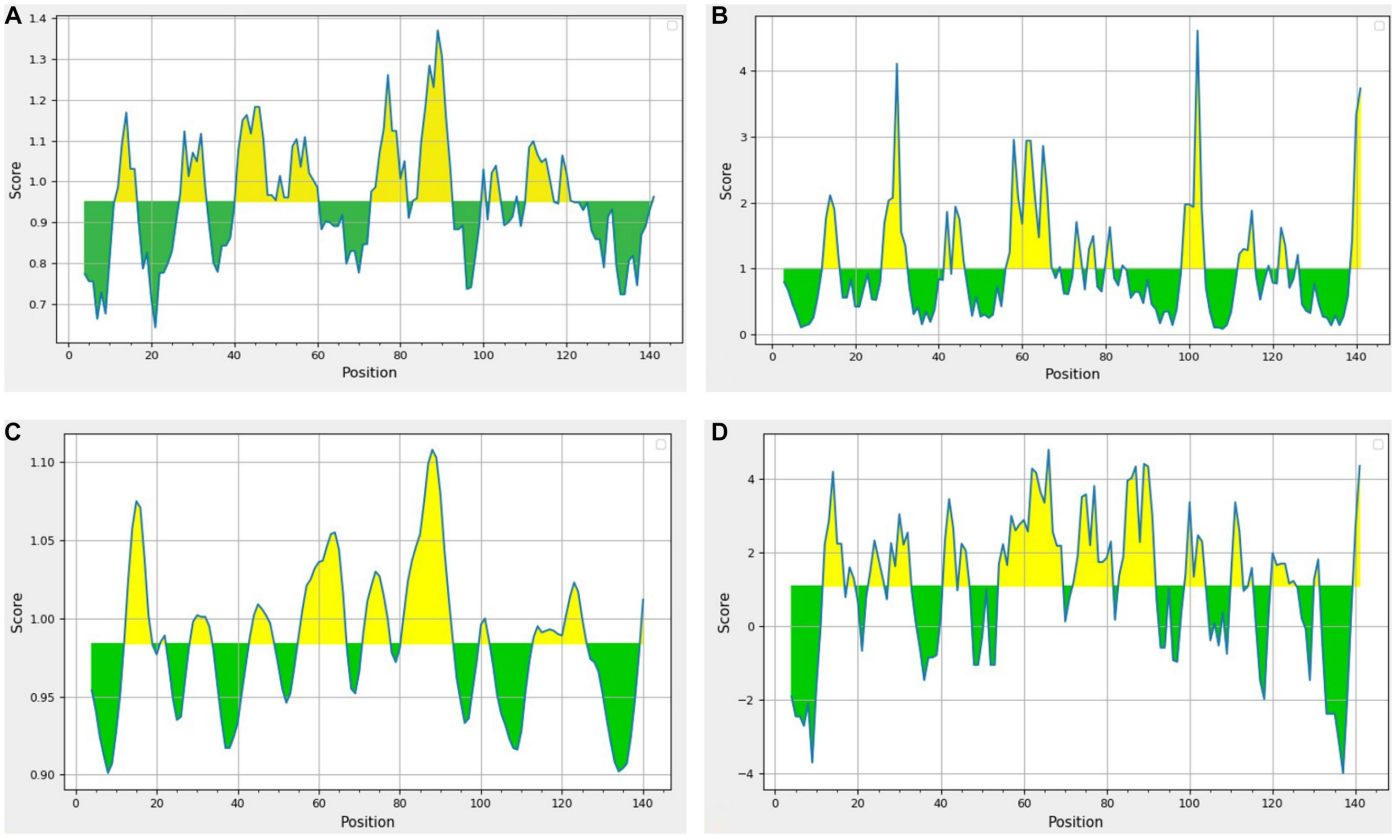
Results of prediction methods **(A)** Chou and Fasman beta-turn (Threshold = 0.951); **(B)** Emini surface accessibility (Threshold = 1.00); **(C)** Karplus and Schulz (Threshold = 0.984); **(D)** Parker’s hydrophilicity (Threshold = 1.087). The area in yellow depicts the score above the threshold and the green depicts the score below the threshold value.

The beta-turn regions in UspA protein were predicted using Chou and Fasman Beta turn prediction and two regions from 75 to 81 (TNAGYPI) and 86 to 92 (SGSGDLG) were identified as constant B turn regions out of seven epitopes above the threshold. Figure 5A shows the predicted peptides for the UspA protein based on sequence position (*x*-axis) and surface probability (*y*-axis). The Emini surface accessibility prediction result predicted two sequences, 27ARPYNA32, where 29P is the surface residue or a residue that is more than 20 Å from water, and 57DMQKRISEETH67, where 59Q is the surface residue. However, the maximum surface probability value calculated by the tool was 4.604 from amino acid position 100 to 105. According to Figure 5B, peptide 106LVVCGH111 (at amino acid positions 106 to 111) had a minimum surface probability value of 0.087. Figure 5C shows the graphical representation of the results anticipated by the flexibility scale approach developed by Karplus and Schulz. The highest flexibility score was 1.108 (heptapeptide: 85 to 91 amino acids). According to results, the sequence of heptapeptide was 85LSGSGDL91, with 88S serving as the surface residue. The more organised portion was assigned a minimum score of 0.901 portion (Figure 5C). Parker’s hydrophilicity prediction predicted linear epitopes based on the hydrophilicity of amino acid residues. The epitope region was located by limiting the window to seven amino acid residues. The results showed that the median value was 1.087, while the maximum and minimum ranged from 4.800 to −4.000. All results that met or exceeded the criteria, as shown in Figure 5D, were likely hydrophilic.

### 3.5 Epitope prediction using other tools

**Figure 6:**
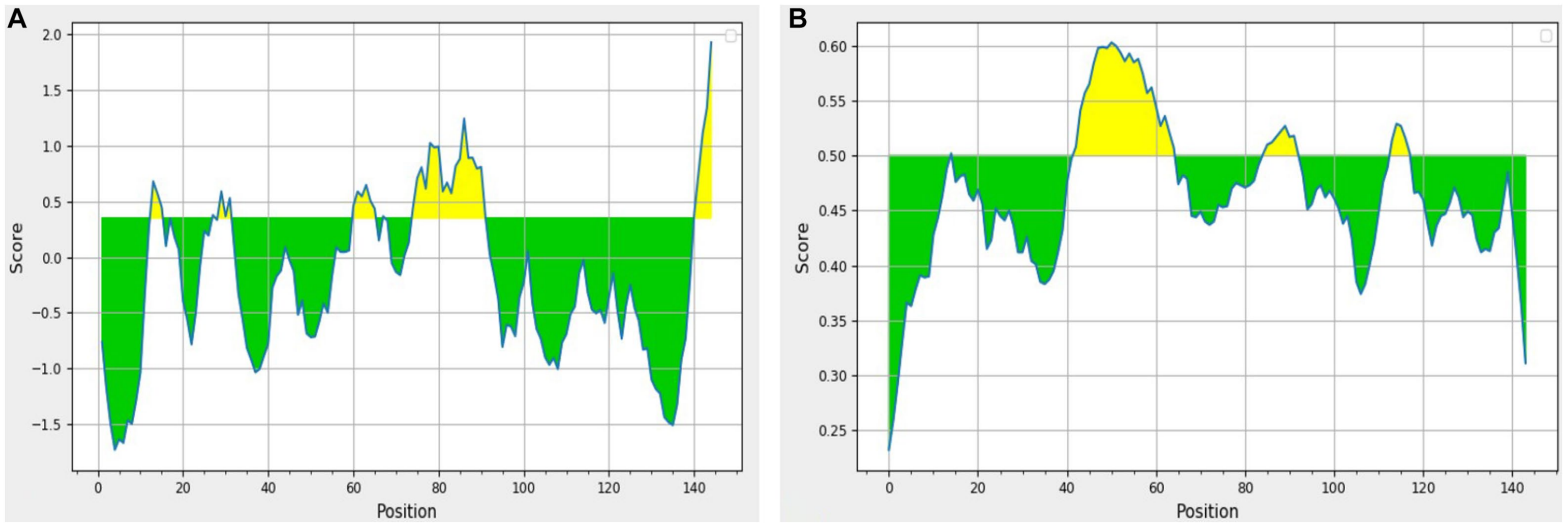
The results predicted by **(A)** BepiPred (Th = 0.350) and **(B)** BepiPred 2.0 (Th = 0.50). The area in yellow depicts the score above the threshold and green depicts the score below the threshold value.

The results obtained using various other tools namely BepiPred linear epitope prediction or BepiPred, BepiPred 2.0 linear epitope prediction, ABCpred, and SVMTrip are summarized in Table 3. Using BepiPred and BepiPred 2.0, residues with a score above the cut-off value (0.5 as the default value) were predicted to be part of the epitope (Figures 6A,B). The trained recurrent neural network result was used by the ABCpred to rank the anticipated B-cell epitopes. The probability of a peptide being an epitope increased with the peptide score. To improve prediction performance, tri-peptide similarity and propensity scores (SVMTriP) were combined with support vector machine (SVM). Two 12-mer peptide sequence namely TGLIDVNLGDMQ and AYKHILIAVDLS, were predicted using SVMTriP.

**Table 3:** B-Cell epitope prediction results using BepiPred, BepiPred 2.0, SVMTrip, and ABCPred.

BepiPred				
No.	Start	End	Peptide	Length
1.	60	65	KRISEE	6
2.	74	91	STNAGYPITETLSGSGDL	18
**BepiPred 2.0**				
3.	43	65	YSDLYTGLIDVNLGDMQKRISEE	23
4.	85	93	LSGSGDLGQ	9
5.	114	118	DFWSK	5
**SVMTrip**				
6.	48	59	TGLIDVNLGDMQ	12
7.	2	13	AYKHILIAVDLS	12

ABCPred				
	Rank	Sequence	Start position	Score
8.	1	SLIHVDVNYS	35	0.78
9.	2	YTGLIDVNLG	47	0.73
10.	3	SMARPYNAKV	25	0.70
11.	4	NLGDMQKRIS	54	0.67
12.	5	ILIAVDLSPE	6	0.65
13.	5	PESKVLVEKA	14	0.65
14.	6	AGYPITETLS	77	0.63

### 3.6 Comparison and suitable peptide candidate

After analysis of different peptides predicted by various methods two peptides namely ARPYNA (6-mer) and YSDLYTGLIDVNLGDMQKRISEE (23-mer) were found as the most suitable peptide candidates for UspA. The peptides used in the comparison along with the scores are listed in Table 4.

**Table 4:** The peptides with their Emini surface accessibility, Kolaskar and Tongaonkar antigenicity, Parker hydrophilicity, and Chou–Fasman beta turn scores.

Peptide	Start	End	Length	Emini surface accessibility score (Th = 1.00)	Kolaskar and Tongaonkar antigenicity score (Th = 1.052)	Parker hydrophilicity prediction score (Th = 1.087)	Chou–Fasman beta turn score (Th = 0.951)
KHILIAVDLS	4	13	10	0.5391	1.113	−1.49275	0.819
ILIAVDLSPE	6	15	10	0.828	1.101	−0.112	0.886
PESKVLVEKA	14	23	10	0.959	1.094	1.465	0.865
ESKVLVEKA	15	23	9	0.828	1.105	1.162	0.831
SMARPYNAKV	25	34	10	1.514	1.03	1.651	0.995
ARPYNA^a^	27	32	6	2.046	1.13	2.069	1.057
AKVSLIHVDVN	32	42	11	0.671	1.114	0.45	0.934
SLIHVDVNYS	35	44	10	0.787	1.117	0.507	0.957
YSDLYTGLIDVNLGDMQKRISEE^a^	43	65	23	1.339	1.141	2.07	1.016
YTGLIDVNLG	47	56	10	0.482	1.138	0.608	1.015
TGLIDVNLGDMQ	48	59	12	0.869	1.018	0.87	1.015
NLGDMQKRIS	54	63	10	1.811	0.961	2.785	1.003
DMQKRISEETH	57	67	11	2.134	0.962	3.332	0.936
KRISEE	60	65	6	2.334	0.96	3.488	0.909
STNAGYPITETLSGSGDL	74	91	18	0.891	0.993	2.8	1.126
TNAGYPI	75	81	7	1.086	0.992	2.463	1.109
AGYPITETLS	77	86	10	0.98	0.998	2.287	1.066
LSGSGDLGQ	85	93	9	0.572	1.013	2.942	1.171
SGSGDLG	86	92	7	0.571	1	3.31	1.222
GQVLVDAI	92	99	8	0.586	1.117	0.06	0.859
KKYDMD	100	105	6	1.91	1.011	1.676	0.976
DLVVCGHH	105	112	8	0.386	1.143	0.769	0.961
DFWSK	114	118	5	1.08	0.998	−0.1688	1.006
VDMLIVPL	132	139	8	0.424	1.115	−2.042	0.795

a Peptides that passed all the four methods.

### 3.7 Structure-based epitope prediction

**Figure 7:**
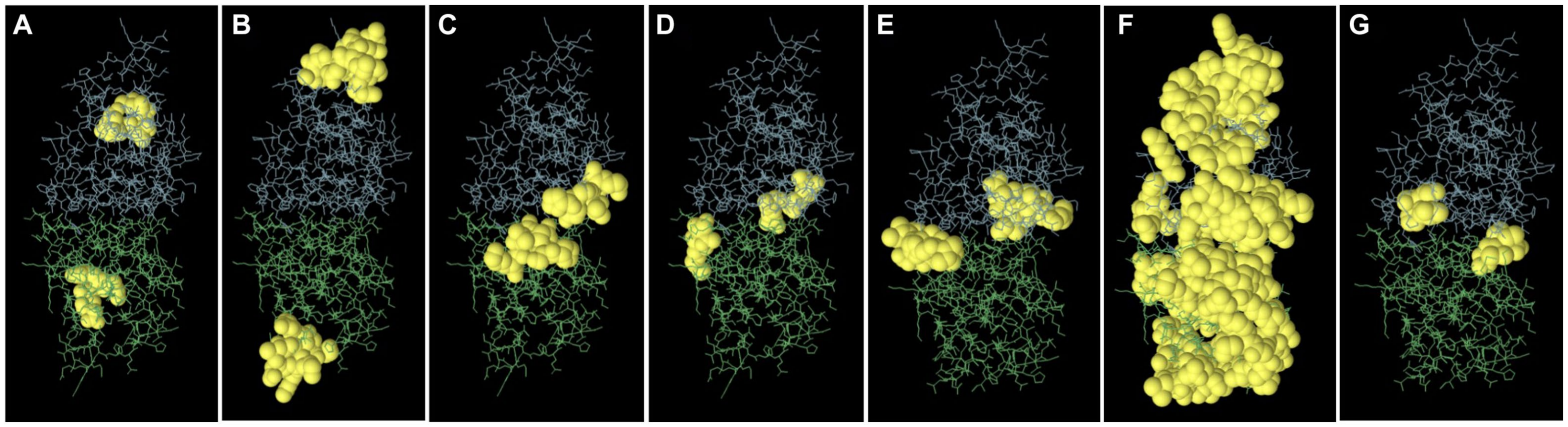
The figure representing discontinues epitopes **(A–G)** of UspA protein of *E. coli*. The bulk of the UspA protein is represented by green sticks for chain A and blue sticks for chain B, while the epitopes are shown as yellow surfaces (spherical balls).

Epitopes were predicted based on protein structure using the ElliPro website tool. Three discontinuous peptides were selected for UspA chain B and four for chain A (score >0.7). The maximum probability of a discontinuous epitope was calculated as 87.7% (protrusion index; PI score: 0.877). Table 5 lists the residues implicated in discontinuous epitopes along with their sequence location, number of residues, and scores, whereas Figure 7 depicts their placements on 3D structures (A through G).

**Table 5:** Discontinuous antigenic epitopes of the UspA protein of *E. coli* predicted using ElliPro web-based tool.

No.	Residues and their positions	Number of residues	Score	3D structure
**For chain A**				
1	N42, Y43, S44, D45, L46	5	0.877	Figure 7A
2	D52, L55, G56, D57, M58, Q59, R61, I62, S63, E64	10	0.783	Figure 7B
3	A2, Y3, K4, P29, Y30, D105	6	0.762	Figure 7C
4	R124, N128, T129	3	0.732	Figure 7D
**For chain B**				
1	Q125, L126, I127, N128, T129, V130, H131, V132	8	0.75	Figure 7E
2	A2, Y3, K4, K17, K22, S25, M26, A27, R28, P29, Y30, N31, A32, D40, V41, N42, Y43, S44, D45, L46, Y47, T48, V53, N54, L55, G56, D57, M58, Q59, K60, I62, S63, E64, T66, H67, H68, T71, E72, T75, N76, A77, G78, Y79, P80, G89, D90, D105, H112, Q113, D114, S117, M120, S121, S122, A123, R124	56	0.677	Figure 7F
3	G110, H111, V137, P138	4	0.626	Figure 7G

## 4 Discussion

APEC is a serious concern to the poultry industry in terms of economic losses, poor welfare of birds, and antimicrobial resistance. Diagnostic and characterization methods for APEC need to be updated with rapid or point-of-care (POC) testing. A total of seven silent mutations were observed in the sequence of non-pathogenic *E. coli* isolate (ECO92LTBW) of the current study. Although these mutations did not alter amino acid sequences of encoded protein directly, they might influence exonic splicing efficiency resulting in change in mRNA processing of genetic information (30). The sequence OM837340 and OM837341 revealed a maximum nucleotide (nt) homology of 99.87%–100% with *E. coli* isolates and a minimum nt homology of 83.73%–84.08% with *Salmonella* species, consistent with previous findings (7). The current study strains exhibited higher closeness (99.25% nt homology) to *Shigella* species, also observed previously for *uspA* gene (31). The DNA hybridization studies in past have also observed that at the species level *Shigella* and *E. coli* are taxonomically indistinguishable (32). On the other side, the *uspA* of *Shigella species* and those of *Salmonella* (retrieved sequences) shared a homology of 85%. Similarly, Wang et al. (33) and Wei et al. (34) proposed that to precisely reflect the evolutionary relationship, a new nomenclature may be decided for *Shigella* and *E. coli*. However, current and above-discussed studies have not employed alternate immunogenic genes for differentiation of aforesaid species. So, further studies using whole genome sequencing may be directed to precisely understand the relatedness of both the species. Tajima’s molecular clock or rate test also signifies the same as there were no divergent sites found on aligning the *uspA* sequences of *Shigella* spp. The null hypothesis that the amount of evolutionary change in two lineages (both *E. coli* isolates and *Shigella*) is equal was not rejected and thus suggests that molecular evolution of *uspA* gene might be occurring at an approximately uniform rate over time. The lower homology among current study isolates as compared to retrieved sequences is also surprising and might be due to different nature (pathogenic or non-pathogenic) or different niche as OM837341 (ECO92LTBW) is of faecal origin and is non-pathogenic and OM837340 (APEC41LFB) is pathogenic isolate recovered from dead bird. This aspect may be further investigated by employing isolates of different origin and pathogenicity. The current study also suggests that entire gene sequence including coding and non-coding region should be used for the sequence homology as protein coding region is almost conserved among different strains of *E. coli* and similar suggestions were also made by other researchers (31). The *uspA* gene has been targeted by various researchers as an identification marker for the *E. coli* using molecular methods (5, 6, 31). At a confidence level of >90%, the core model was extremely accurate with 2–4 Å RMSD from the native structure, according to the SWISS Model. The current study sequence and template employed shared 67.86% identity, which implies an extraordinarily high accuracy model. The MolProbity score for current study’s UspA protein is 1.31. This score is a combined protein quality score which indicates the crystallographic resolution at which a good-quality model is anticipated (17). The low number indicates a good quality model of protein structure.

Kolaskar and Tongaonkar’s technique predicted six antigenic peptides for UspA protein. This method uses the physicochemical characteristics of amino acid residues that frequently appear in experimentally determined antigenic epitopes. According to earlier studies, this approach has an experimental accuracy rate of 75% (20). Our findings are in consensus with Mishra et al. (7), where the researchers predicted seven peptides for UspA protein using diarrheagenic *E. coli* isolates. Based on high score among the predicted peptides, “KHILIAVDLS” might be a potential candidate for the diagnostic assay or vaccine development. This finding is also supported by the previous investigation by Mishra et al. (7). The Chou–Fasman algorithm predicted two constant beta turn regions at 75–81 and 86–92 amino acid positions. The immunodominant region of a protein is located in beta turn region and protein’s beta twists are often hydrophilic and surface-accessible, and they significantly contribute to the development of antigenicity (18). However, no comparable studies exist for epitope prediction using *usp*A in *E. coli*, beta turn prediction is reported as the most frequently used propensity (27) out of several propensities or models in use.

The surface probability of a hexapeptide greater than 1.0 (threshold), as suggested by Emini et al. (19), indicates that the sequence has a higher likelihood of being detected on the surface. The highest surface probability that could be obtained for the sequence 100KKYDMD105 was 4.604, however, its comparison with other propensities (beta turn, hydrophilicity, etc.) might not prove it a suitable candidate. However, two other peptides namely 27ARPYNA32, and 57DMQKRISEETH67 might prove better candidates. The epitope with more surface probabilities may prove a crucial candidate for an *E. coli* peptide vaccine. Using Karplus and Schulz’s flexibility scale, for peptide 85LSGSGDL91, the maximum flexibility score was 1.108. This approach generates the B factor or atomic temperature factor which represents vibrational motion of atoms within structure. A lower B factor value represents a well-organized structure, while a greater value indicates a fluid structure (20). The creation of an *E. coli* diagnostic assay may benefit from the anticipated flexibility of the UspA protein. The Parker’s prediction of an amino acid’s hydrophilicity predicted seven regions which are based on retention duration of peptide on a reversed phase column in high performance liquid chromatography (HPLC) and values that fall within and above the threshold are probably hydrophilic (22).

BepiPred 2.0 predicted two whereas BepiPred predicted three peptides as suitable epitope regions. BepiPred 2.0 predicts B-cell epitopes from a protein sequence using a random forest algorithm trained on epitopes and non-epitope amino acids that it identifies from crystal structures, whereas BepiPred predicts the location of B-cell epitopes using a hidden Markov model and a propensity scale method (23, 24). These models were also previously used by Elhag et al. (28) for prediction of antigenic epitopes for proposing a vaccine against *Pseudomonas aeruginosa*. Similarly, another tool ABCpred predicted six B cell epitopes. This tool works on the scores that the trained recurrent neural network assigned to each peptide. The greater the peptide’s score, the more likely it is to be an epitope. Antigenic epitope prediction by ABCpred is accurate up to 65.93% (25).

Although 24 different B-cell epitopes were predicted by various tools and/or models used in the study, the results were compared based on the scores of four methods as described in the literature (27, 28). The analysis revealed two most suitable candidates namely APYRNA and YSDLYTGLIDVNLGDMQKRISEE. These predicted peptides may be utilized for further investigations and development of diagnostic assays.

Eight discontinuous peptides were predicted in the current study using ElliPro. ElliPro distinguishes epitopes based on protein-antibody interactions. It establishes a correlation between a protein’s solvent accessibility, antigenicity, and flexibility. The score, commonly known as the protrusion index (PI) value, displays the proportion of protein atoms engaged in antibody binding that protrude beyond the molecular bulk (ellipsoid). For the current study, 87.7% was shown to be the highest chance of a discontinuous epitope (PI score: 0.877). In comparison to other tools, ElliPro has been tested on common data of conformational antibody-protein complexes and is more user-friendly (29). There is no comparable studies using UspA protein to validate the results obtained. However, this aspect may be further investigated to draw conclusive evidence and/or develop an assay or diagnostic method.

Moreover, contact binding and epitope mapping of immunodominant epitopes on the universal stress protein could define functional characteristics with regard to pathogenicity. UspA family appears to be crucial for the bacteria to support cellular defense mechanisms and oxidative stress (3, 4). Many immunological assays can target the *uspA* gene, and it can also be considered as a potential candidate for multiple subunit vaccines and/or as a diagnostic marker. Based on the degree of methodological correctness, the predicted peptides might be a putative candidate for use as an epitope in diagnostics. The inclusion of other peptides of virulence factors discovered in pathogenic *E. coli* may increase the efficacy of these as a suitable epitope for the development of multiple subunit vaccines. Also, by using lateral flow assay techniques, these peptides could serve as an antigen for the detection of certain antibodies against *E. coli*.

## 5 Conclusion

The two promising applications of B-cell epitope prediction are development of vaccine and diagnostics. Antigenic epitope-based peptides appear to be appealing candidates for diagnostic, preventive, and therapeutic vaccinations. The current study utilizes UspA protein to predict the antigenic epitopes and thus select the most suitable candidates. A total of 24 linear epitope peptides and seven discontinuous peptides were precited using various online tools which on further analysis yielded two most suitable candidates viz., APYRNA and YSDLYTGLIDVNLGDMQKRISEE. These peptides might prove as potential candidates for peptide based diagnostic assay for *E. coli*. Further applied research might be directed to develop these anticipated peptides into diagnostic assay/method. The phylogenetic analysis and molecular clock hypothesis of the *uspA* gene’s coding region in the current study showed that *Shigella* and *E. coli* share huge similarities concerning *uspA* gene. The 3D structure of UspA protein validated in the current study may be utilized further for molecular docking or protein interactions studies.

## Data availability statement

The original contributions presented in the study are included in the article/supplementary material, further inquiries can be directed to the corresponding author/s.

## Author contributions

KG and NJ: conceptualization. KG: methodology and Writing—original draft preparation. KG and DM: formal analysis and investigation. KG, DM, AP, RK, and NJ: writing—review and editing. DM, RK, AP, and NJ: supervision. All authors contributed to the article and approved the submitted version.

## References

[1] Acharya V. Urinary tract infection—a dangerous and unrecognised forerunner of systemic sepsis. J Postgrad Med. (1992) 38:52. Available at: https://www.jpgmonline.com/article.asp?issn=0022-4 PMID: 1432825

[2] Grakh K Mittal D Prakash A Jindal N. Characterization and antimicrobial susceptibility of biofilm-producing avian pathogenic *Escherichia coli* from broiler chickens and their environment in India. Vet Res Commun. (2022) 46:537–48. doi: 10.1007/S11259-021-09881-5, PMID: 35112272

[3] Siegele DA. Universal stress proteins in *Escherichia coli*. J Bacteriol. (2005) 187:6253–4. doi: 10.1128/JB.187.18.6253-6254.2005, PMID: 16159755 PMC1236659

[4] Nachin L Nannmark U Nyström T. Differential roles of the universal stress proteins of *Escherichia coli* in oxidative stress resistance, adhesion, and motility. J Bacteriol. (2005) 187:6265–72. doi: 10.1128/JB.187.18.6265-6272.2005, PMID: 16159758 PMC1236625

[5] Chen J Griffiths MW. PCR differentiation of *Escherichia coli* from other Gram-negative bacteria using primers derived from the nucleotide sequences flanking the gene encoding the universal stress protein. Lett Appl Microbiol. (1998) 27:369–71. doi: 10.1046/J.1472-765X.1998.00445.X, PMID: 9871356

[6] Osek J. Multiplex polymerase chain reaction assay for identification of enterotoxigenic *Escherichia coli* strains. J Vet Diagn Investig. (2001) 13:308–11. doi: 10.1177/104063870101300405 11478602

[7] Mishra AK Deepak Singh D Kumarsen G Gupta G Sharma N Kumar N. *UspA* gene based characterization of *Escherichia coli* strains isolated from different disease conditions in goats. J Anim Res. (2017) 7:1123–8. doi: 10.5958/2277-940X.2017.00168.1

[8] Nystrom T Neidhardt FC. Cloning, mapping and nucleotide sequencing of a gene encoding a universal stress protein in *Escherichia coli*. Mol Microbiol. (1992) 6:3187–98. doi: 10.1111/J.1365-2958.1992.TB01774.X, PMID: 1453957

[9] Pandey S Malviya G Chottova DM. Role of peptides in diagnostics. Int J Mol Sci. (2021) 22:8828. doi: 10.3390/IJMS22168828, PMID: 34445532 PMC8396325

[10] Kvint KL Nachin AD Nystrom T. The bacterial universal stress protein: function and regulation. Curr Opin Microbiol. (2003) 6:140–5. doi: 10.1016/S1369-5274(03)00025-0 12732303

[11] Zhang W Xiong Y Zhao M Zou H Ye X Liu J. Prediction of conformational B-cell epitopes from 3D structures by random forests with a distance-based feature. BMC Bioinformatics. (2011) 12:1–10. doi: 10.1186/1471-2105-12-341/FIGURES/5 21846404 PMC3228550

[12] Kumar S Stecher G Li M Knyaz C Tamura K. MEGA X: molecular evolutionary genetics analysis across computing platforms. Mol Biol Evol. (2018) 35:1547–9. doi: 10.1093/MOLBEV/MSY096, PMID: 29722887 PMC5967553

[13] Tamura K Stecher G Kumar S. MEGA11: molecular evolutionary genetics analysis version 11. Mol Biol Evol. (2021) 38:3022–7. doi: 10.1093/molbev/msab120, PMID: 33892491 PMC8233496

[14] Tajima F. Simple methods for testing the molecular evolutionary clock hypothesis. Genetics. (1993) 135:599–607. doi: 10.1093/GENETICS/135.2.599, PMID: 8244016 PMC1205659

[15] Hall TA. BioEdit: a user-friendly biological sequence alignment editor and analysis program for Windows 95/98/NT. In Nucleic acids symposium series (1999) 41:95–98. Available at: https://www.academia.edu/download/29520866/1999hall1.pdf

[16] Waterhouse A Bertoni M Bienert S Studer G Tauriello G Gumienny R. SWISS-MODEL: homology modelling of protein structures and complexes. Nucleic Acids Res. (2018) 46:W296–303. doi: 10.1093/nar/gky427, PMID: 29788355 PMC6030848

[17] Gooch JW. Ramachandran plot. In: Encyclopedic dictionary of polymers. Springer Science & Business Media. (2011). 1:919–9.

[18] Chou PY Fasman GD. Prediction of the secondary structure of proteins from their amino acid sequence. Adv Enzymol Relat Areas Mol Biol. (1978) 47:45–148. doi: 10.1002/9780470122921.CH2 364941

[19] Emini EA Hughes J Perlow DS Boger J. Induction of hepatitis a virus-neutralizing antibody by a virus-specific synthetic peptide. J Virol. (1985) 55:836–9. doi: 10.1128/jvi.55.3.836-839.1985, PMID: 2991600 PMC255070

[20] Karplus PA Schulz GE. Prediction of chain flexibility in proteins—a tool for the selection of peptide antigens. Naturwissenschaften. (1985) 72:212–3. doi: 10.1007/BF01195768

[21] Kolaskar AS Tongaonkar PC. A semi-empirical method for prediction of antigenic determinants on protein antigens. FEBS Lett. (1990) 276:172–4. doi: 10.1016/0014-5793(90)80535-Q, PMID: 1702393

[22] Parker JMR Guo D Hodges RS. New hydrophilicity scale derived from high-performance liquid chromatography peptide retention data: correlation of predicted surface residues with antigenicity and X-ray-derived accessible sites. Biochemistry. (1986) 25:5425–32. doi: 10.1021/bi00367a013, PMID: 2430611

[23] Larsen JEP Lund O Nielsen M. Improved method for predicting linear B-cell epitopes. Immunome Res. (2006) 2:2. doi: 10.1186/1745-7580-2-2, PMID: 16635264 PMC1479323

[24] Jespersen MC Peters B Nielsen M Marcatili P. BepiPred-2.0: improving sequence-based B-cell epitope prediction using conformational epitopes. Nucleic Acids Res. (2017) 45:W24–9. doi: 10.1093/nar/gkx346, PMID: 28472356 PMC5570230

[25] Saha S Raghava GPS. Prediction of continuous B-cell epitopes in an antigen using recurrent neural network. Proteins. (2006) 65:40–8. doi: 10.1002/PROT.21078 16894596

[26] Yao B Zhang L Liang S Zhang C. SVMTriP: a method to predict antigenic epitopes using support vector machine to integrate tri-peptide similarity and propensity. PLoS One. (2012) 7:e45152. doi: 10.1371/JOURNAL.PONE.0045152, PMID: 22984622 PMC3440317

[27] Su H Pal NR Lin L Chung F. Identification of amino acid propensities that are strong determinants of linear B-cell epitope using neural networks. PLoS One. (2012) 7:e30617. doi: 10.1371/journal.pone.0030617, PMID: 22347389 PMC3275595

[28] Elhag M Alaagib RM Ahmed NM Abubaker M Haroun EM Albagi SO. Design of epitope-based peptide vaccine against *Pseudomonas aeruginosa* fructose bisphosphate aldolase protein using immuno informatics. J Immunol Res. (2020) 2020:2020. doi: 10.1155/2020/9475058, PMID: 33204735 PMC7666636

[29] Ponomarenko J Bui HH Li W Fusseder N Bourne PE Sette A. ElliPro: a new structure-based tool for the prediction of antibody epitopes. BMC Bioinformatics. (2008) 9:514. doi: 10.1186/1471-2105-9-514, PMID: 19055730 PMC2607291

[30] Antonarakis S Cooper D. Human gene mutation in inherited disease: molecular mechanisms and clinical consequences In: Rimoin D Pyeritz R Korf B, editors. Principles and practices of medical genetics. Emery and Rimoin’s Principles and Practice of Medical Genetics. Elsevier: Academic Press (2013). 1–48. doi: 10.1016/B978-0-12-383834-6.00007

[31] Chen J. *uspA* of *Shigella sonnei*. J Food Prot. (2007) 70:2392–5. doi: 10.4315/0362-028X-70.10.2392, PMID: 17969624

[32] Brenner DJ Fanning GR Steigerwalt AG Orskov I Orskov F. Polynucleotide sequence relatedness among three groups of pathogenic *Escherichia coli* strains. Infect Immun. (1972) 6:308–15. doi: 10.1128/IAI.6.3.308-315.1972, PMID: 4564889 PMC422532

[33] Wang L Qu W Reeves PR. Sequence analysis of four *Shigella boydii* O-antigen loci: implication for *Escherichia coli* and *Shigella* relationships. Infect Immun. (2001) 69:6923–30. doi: 10.1128/IAI.69.11.6923-6930.2001, PMID: 11598067 PMC100072

[34] Wei J Goldberg MB Burland V Venkatesan MM Deng W Fournier G. Complete genome sequence and comparative genomics of *Shigella flexneri* serotype 2a strain 2457T. Infect Immun. (2003) 71:2775–86. doi: 10.1128/IAI.71.5.2775-2786.2003, PMID: 12704152 PMC153260

